# Salt‐induced nutritional and metabolic shifts in halophytes: implications for food security

**DOI:** 10.1002/jsfa.70212

**Published:** 2025-09-26

**Authors:** Giulia Atzori, Pedro Garcia Caparros, Antonella Castagna, Luisa Custódio, Valentina Lazazzara, Claus Krogh Madsen, Felicia Menicucci, Maria João Rodrigues, Katalin Solymosi, Jose Ramon Acosta Motos

**Affiliations:** ^1^ Institute of Sustainable Plant Protection (CNR‐IPSP) National Research Council of Italy Florence Italy; ^2^ Higher Engineering School Univeristy of Almeria Almeria Spain; ^3^ Department of Agricultural, Food and Agro‐Environmental Sciences University of Pisa Pisa Italy; ^4^ Centre of Marine Sciences (CCMAR/CIMAR LA) University of Algarve, Faculty of Sciences and Technology Faro Portugal; ^5^ Department of Agroecology Aarhus University Slagelse Denmark; ^6^ Department of Plant Anatomy, Institute of Biology, Faculty of Science ELTE Eötvös Loránd University Budapest Hungary; ^7^ Plant Biotechnology for Food and Agriculture Group, Universidad Católica San Antonio de Murcia Murcia Spain

**Keywords:** saline agriculture, halophytes, potential new crops, salt‐induced plant metabolism, elements accumulation, nutritional and anti‐nutritional compounds

## Abstract

Plant species vary in their response to salinity: some crops show a degree of salt tolerance, while halophytes – whether wild or cultivated – are characterized by a high capacity to thrive under saline conditions. Halophytes are considered a source of valuable secondary metabolites with potential economic value, yet they might also produce secondary metabolites posing nutritional challenges for human consumption. Moreover, halophytes often accumulate high concentrations of sodium and chlorides. However, a clear understanding of how salinity modulates the production of both the beneficial and non‐nutritional factors is limited because widely variable in relation to specific environments and species. This review, written in the framework of the COST Action SUSTAIN (CA22144) on the sustainable use of salt‐affected land, explores the main potential and challenges associated with the introduction of halophytes in the human diet through a revision of the effects of salinity on salt tolerant plants' secondary metabolism and mineral elements' accumulation. Moreover, the potential antioxidant, anti‐inflammatory and anticancer properties of halophytes are discussed. © 2025 The Author(s). *Journal of the Science of Food and Agriculture* published by John Wiley & Sons Ltd on behalf of Society of Chemical Industry.

## INTRODUCTION

The global population is projected to exceed 9.5 billion people by 2050.^1^ The challenges of attaining sustainable crop growth and food security are, and will likely continue to be, exacerbated by climate change and extreme weather fluctuations, and by the growing population itself. Indeed, anthropogenic activities have exacerbated the impacts of climate change on ecosystems.^2^ Among those impacts, increasing soil salinity has a direct effect on our main crops, which cannot withstand significant salinization. The risk of soil salinization is expected to vary across latitudes, with Mediterranean regions being particularly susceptible due to rising temperatures, increased evapotranspiration and evaporation rates, and reduced precipitation. These climatic shifts are likely to result in diminished water availability, lower reservoir levels, and a more irregular distribution of rainfall.^3^, ^4^, ^5^

Soil salinity is a growing threat to global food security, limiting plant growth by altering water uptake dynamics, disrupting essential ion gradients, and inducing oxidative damage at the cellular level.^6^, ^7^, ^8^ Salt stress affects plants at various physiological and molecular levels, leading to reduced germination, inhibited photosynthesis, and metabolic dysregulation.^9^, ^10^, ^11^ To survive in saline environments, plants must develop intricate defence mechanisms that include morphological, physiological, and biochemical modifications. Halophytes, naturally adapted to high salinity, and some glycophytes with enhanced salt tolerance, activate a suite of mechanisms.^12^, ^13^ These morphological, physiological, and biochemical adaptations enable them to survive, grow, and even thrive in environments where most plants would suffer significant damage.^14^, ^15^

In this review we address the main advantages and challenges associated with the use of halophytes and salt tolerant crops for human diet, correlating salinity to the accumulation or the production of beneficial metabolites as well as non‐nutritional ones. Moreover, the potential uses of antioxidant, anti‐inflammatory and anticancer properties of halophytes are discussed.

## ELEMENTS ACCUMULATION IN THE EDIBLE PLANT TISSUES

### Macroelements’ and microelements' accumulation

Mineral nutrients play a vital role in all plant processes including emergence, development, productivity, metabolism and protection.^16^ They can be characterized as macronutrients, for example, calcium (Ca), magnesium (Mg), sulphur (S), nitrogen (N), potassium (K), and phosphorus (P), and micronutrients, for example, iron (Fe), boron (B), copper (Cu), manganese (Mn), chlorine (Cl), nickel (Ni), molybdenum (Mo), cobalt (Co), and zinc (Zn), based on their relative concentration in the plant tissue. Other elements, including silicon (Si), selenium (Se), and sodium (Na), are referred to as beneficial elements: they promote plant growth and are essential for some but not for all higher plant species. However, for each element and plant species, the roles played by these last nutrients vary.^17^

While mineral nutrients are essential to crops and can protect plants against both abiotic and biotic stresses,^18^ the same nutrients are important in the nutrition of those who consume the plants,^19^ including humans. Of the at least 49 nutrients required by humans to meet their metabolic needs, 22 are mineral elements and their inadequate consumption can result in adverse metabolic disturbances.^20^, ^21^ Over three billion people are afflicted with mineral element malnutrition, linked not only to an inadequate quantity of food but also to its quality: Ca, Mg and Cu deficiencies, for example, are common in both developed and developing countries.^22^

The relation between salinity and mineral nutrition of crops is complex. Crop performance might be negatively affected by nutritional disorders caused by the effect of salinity on nutrient availability, competitive uptake, transport or partitioning within the plant.^23^ In a non‐saline context, Na is described as a non‐essential but beneficial nutrient^24^, ^25^, ^26^ and Cl is described as an essential micronutrient,^27^, ^28^ and recently, Cl has been described as a beneficial macronutrient, improving water balance and plant productivity.^29^, ^30^ However, in a context of salinity, crop performance may be negatively affected by salinity,^23^ especially cultivated crops, that are basically glycophytes.^31^

The ion uptake mechanism and their pattern of accumulation in various plant parts are very important in distinguishing salt‐tolerant and salt‐sensitive genotypes.^32^ Even if numerous interactions between salinity and nutrients occur simultaneously, whether they ultimately affect crop yield or quality depends upon the salinity level and composition of salts, the crop species, the specific nutrient and environmental factors.^23^ Most of the studies reported in scientific literature deal either with non‐edible crops, that is, Ashraf and Ahmad^32^ compare salt‐tolerant and salt‐sensitive lines of cotton (*Gossypium hirsutum* L.), or with non‐edible portions of edible crops, that is, the study on salt‐tolerant and salt‐sensitive cultivars of rice (*Oryza sativa* L.) of Kumar *et al*.^33^ investigating the elements accumulation in the shoots and in the roots but not in the seeds, and the experiment of Ali *et al*.^34^ comparing two barley (*Hordeum vulgare* L.) genotypes differing in salt tolerance and assessing K, Mg, Ca, Fe, Zn, Mn and Cu accumulation only in shoot and root tissues. More data are instead available for leafy vegetables: a study confronting glycophytes characterized by different salt tolerance (i.e., lettuce, chard and chicory) assessed different patterns of mineral elements accumulation: Na increased in all species, Ca accumulation was not affected in lettuce and chicory, while it increased in chard – the species with the highest salt tolerance among the three – when treated with the higher seawater concentration.^35^ Different patterns of accumulation were also observed comparing lettuce plants grown using different saline solutions, that is, Hoagland solution supplemented with seawater or with sodium chloride (NaCl) solution: whereas Ca dropped compared to the control in both saline solution cases, K decreased compared to non‐saline conditions only when plants were grown with the NaCl solution, and not with seawater.^36^

When focusing on halophytes, salinity often increases the accumulation of several mineral elements. An increase in Na and a decrease in K is often observed, with the accumulation of Na coming at the cost of K and resulting in high Na/K ratios. However, it has also been shown that a cytosolic Na/K ratio smaller than 1 does not seem generally essential for halophytes tolerance to high salinity conditions.^37^ In the edible halophyte *Mesembryanthemum crystallinum* L., together with Na, also the Ca concentration rose in salt‐treated leaves compared to the control, whereas K decreased.^38^ A similar pattern was observed in another edible halophyte, *Tetragonia tetragonioides* (Pall.) Kuntze: together with a Na increase and a K decrease, salt‐treated plants accumulated higher Mg, Cu and Zn compared to plants grown under non‐saline conditions, both at the shoot and root level.^39^ Similarly, the edible *Salsola soda* L. showed an increase in Na and a decrease in K, together with an increase in Fe, Mg, Mn, Cu, Zn, and Mo when grown under saline conditions compared to control plants.^40^ Such increased accumulation of mineral elements is important because of the current diet of over two‐thirds of the world's population lacking one or more essential mineral elements. However, specific attention needs to be addressed to the Na and Cl components of such potential mineral enrichment.

Important differences in the accumulation pattern of Na and Cl are present even among halophytes,^41^ with two subgroups^42^: (i) euhalophytes, accumulating large amounts of salts in their organs not being damaged, whilst being stimulated at certain concentrations; (ii) miohalophytes, characterized by species‐specific thresholds of salinity tolerance.^40^ Back in 1984, Glenn and O'Leary^43^ evaluated the growth rates and levels of Na, K, Mg, Ca and water in 20 dicotyledonous halophytes grown along a salinity gradient from freshwater to 720 mmol L^−1^ NaCl. Ten test edible species exhibited growth stimulation by 180 mmol L^−1^ NaCl and were classified as euhalophytes. Ten others showed their best growth on freshwater and were classified as miohalophytes. Sodium accumulated in all the studied halophytes but to a significantly higher concentration in euhalophytes compared to miohalophytes. Water content and cation accumulation in euhalophytes appeared to be coordinated to produce a constant osmotic potential gradient within the shoot tissues relative to the external salinity. An increase in Na and Mg and a decrease in K was assessed in almost all the analysed halophytes – with a few exceptions increasing K accumulation under saline conditions, that is, *Haplopappus venetus* (HBK) Blake (Asteraceae) and *Helianthus annuus* L. subsp. *lenticularis* (Doug X Lindl.) Cookery (Asteraceae), this last species having seeds relevant for human consumption. On the opposite, the accumulation of Ca showed higher variability among the tested species and among the different salinity levels.^43^ As described in detail earlier, changes in Ca levels in the cytosol are responsible for mediating signals to different target groups, for example, receptors, scavenger systems, and phytohormones,^44^ and uncertainties remain about the exact linkage between Ca and abscisic acid (ABA) related to sensing in the roots and guard cells.^45^ Calcium signalling is highly complex, relying on interactions with various chemicals and mechanisms, and modern gene‐editing techniques could help elucidate transduction pathways and genes associated with salt stress.^46^

### Heavy metals accumulation

The use of halophytes as edible plants requires careful consideration, as their ability to survive under extreme conditions also enables them to accumulate high levels of heavy metals. Several species within the genus *Salicornia* have demonstrated significant heavy metal uptake capabilities, that is, *Salicornia europaea* has been used in the restoration of salt marshes due to its ability to absorb arsenic (As), cadmium (Cd), Cu, lead (Pb) and mercury (Hg).^47^ Similarly, *Salicornia ramosissima* has been used for the decontamination of areas with high accumulation of Cd, As and Pb,^48^ *Salicornia fruticosa* has been studied for its Cd uptake potential,^49^ *Salsola soda* accumulates Se^50^ and boron (B),^51^ while *Inula crithmoides* showed Cd bioaccumulation.^52^ Extensive research has also documented the phytoremediation capabilities of *Portulaca oleracea*: Negi^53^ reported that this species is an efficient bioaccumulator of As, Cd, Pb, and Cu; Tiwari *et al*.^54^ highlighted its hyperaccumulation capacity for Cd, chromium (Cr), and As; Ozyigit *et al*.^55^ documented its ability to accumulate Cd, Cu and Zn. Further studies have demonstrated its uptake of Zn, Pb,^56^ Ni and Cd.^57^ Similarly, *Sarcocornia perennis* can accumulate Cd and Pb^58^ as well as Zn and Cu.^59^ *Cakile maritima* uptakes several heavy metals, such as Cd^60^ and Ni.^61^ *Mesembryanthemum crystallinum* has been identified as an edible halophyte with significant heavy metal uptake potential,^62^ particularly for Ni,^63^ Cd and Cr.^64^ Overall, while halophytes hold considerable promise as alternative edible crops and phytoremediation agents, their potential heavy metal accumulation necessitates rigorous monitoring and site‐specific risk assessments to ensure food safety and environmental sustainability.^65^ Halophytes' heavy metal concentration should be analysed for the specific production site and then related to safety thresholds before introducing the product for alimentary purposes. Figure 1 depicts a schematic description of the responses of glycophytes and halophytes to salinity.

**Figure 1:**
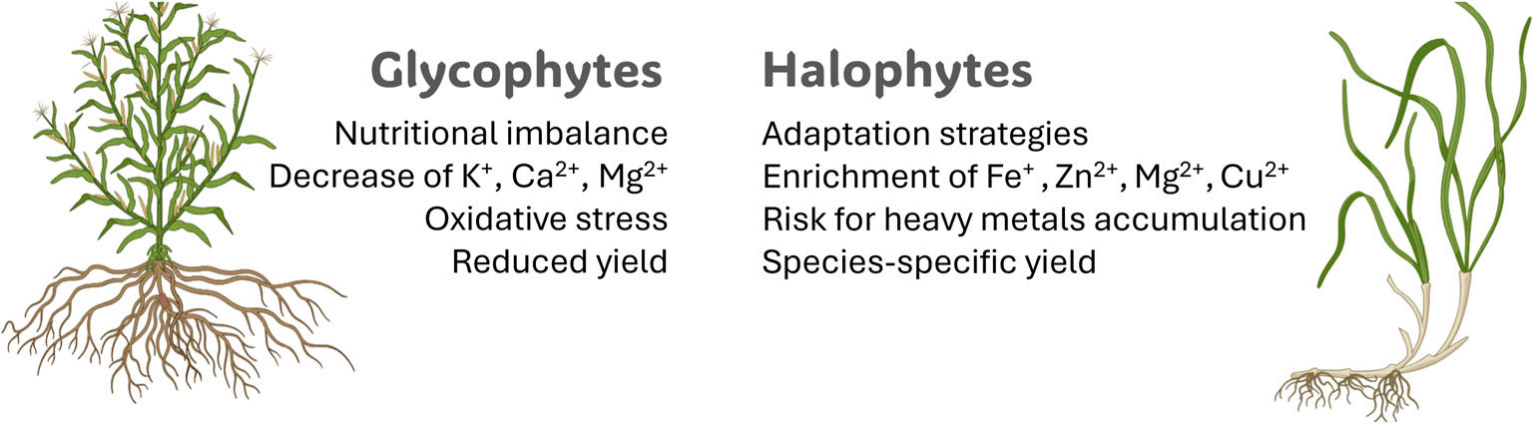
Comparative responses of glycophytes and halophytes to salinity.

## SECONDARY METABOLITES IN THE EDIBLE PLANT TISSUES

### Metabolites with nutritional or nutraceutical positive properties

The production of a plethora of bioactive compounds is part of halophytes' biochemical mechanisms to adapt to extreme saline environments. The consumption of edible halophytes provides thus a considerable nutrient intake.^66^ Interestingly, compounds important for plant defence under adverse environmental conditions – thus typically up‐regulated under saline conditions – play an important role in human nutrition.

#### Polyphenols

Polyphenols constitute a very large family, subdivided into four main classes (e.g., flavonoids, lignans, phenolic acids and stilbenes), characterized by the presence of multiple phenolic groups conferring strong antioxidant properties.^67^, ^68^ Antioxidants are known to play a key role in the prevention of the oxidative stress damage caused by reactive oxygen species (ROS) excess, which is strictly associated with many health injuries.^69^ Hence, the consumption of antioxidant‐enriched food is strongly recommended, and such products are nowadays highly demanded in the market.

Edible halophytes find their place within this framework. Indeed, stress‐related metabolites are generally more concentrated in halophytes than in glycophytes. Boestfleisch *et al*.^70^ investigated the relation between salinity and the concentration of several secondary metabolites with antioxidant properties, among which total polyphenols and flavonoids. The study considered different halophytes showing that it was possible to maximize plant antioxidant capacity by altering the saline growing environment. Particularly, this was observed in *Tripolium pannonicum*, *Plantago coronopus*, *Lepidium latifolium* and *Salicornia europaea*. The authors also found different responses to salinity in relation to plant development state,^70^ as also observed in moderately salt tolerant *Kalanchoe daigremontiana*.^71^ Another study conducted on date palm (*Phoenix dactylifera*) reported that leaves and roots of the salt‐tolerant cultivar exhibited a significantly higher production of polyphenols and flavonoids, indicating a strict relation between salinity tolerance and antioxidation.^72^ Similarly, different ecotypes of the edible halophytes *Salicornia persica* and *Sarcocornia fructicosa* were found to exhibit high total polyphenol content (TPC) values.^73^, ^74^ The search for new natural antioxidants is of utmost importance worldwide, with halophytes being among these potential sources.^75^

#### Chlorophylls

Chlorophylls (Chls) and carotenoids are major photosynthetic pigments synthesized by plastids and accumulated in high amounts in plants. Both Chls and carotenoids are widely used by the food industry as natural food colourings and because of their pharmaceutical and nutraceutical applications,^76^, ^77^ including antimicrobial activity and antioxidant properties.^78^ Most data on the effects of salinity on Chl content in crops focus on non‐edible portions of the tested plants, exception made for the leafy vegetables, that is, lettuce,^79^ sweet basil^80^ and spearmint^81^ all decreasing their Chl concentration upon exposure to salt stress. The scientific evidence on halophytes, such as *Thelungiella salsuginea*, observed increased Chl content under saline conditions,^82^ and *Paspalum vaginatum*, where the Chl content remained either unchanged or at higher salt concentrations declined.^83^ A study on approximately 100 rice varieties showed that depending on the cultivar, the Chl content either declined, remained unchanged or increased.^84^ The complexity and species‐specificity of the effect of salt stress on Chl is further outlined by another study, in which analyses of the stress response of 25 bread and durum wheat cultivars indicated that Chl content and shoot sap K content were promising tools to screen NaCl tolerance.^85^

#### Carotenoids

Carotenoids are widely recognized nutraceuticals. The consumption of carotenoid‐rich products is often recommended for their health benefits, including antioxidant activity and hypolipidemic effects resulting in positive effects for cardiovascular health.^76^ They also show anti‐inflammatory, immune function enhancing and neuroprotective effects improving cognitive function, and are involved in the prevention of various cancer types. However, carotenoids are best known for their role in vision as provitamin A and to protect against age‐related macular degeneration. Carotenoids are also widely used as natural colorants,^76^, ^86^ and as nutritional supplements in functional foods.

Under salt stress, literature data report decreased carotenoid concentration in salt stressed plants for several species, even if such data most of the time do not necessarily refer to plants' edible portions. However, in sweet basil plants carotenoid content was enhanced by salinity.^80^ Similarly, carotenoid content, especially that of lycopene,^87^ lutein, *β*‐carotene, and violaxanthin,^88^ was significantly increased by salt stress in tomatoes,^87^ although fruit size decreased under salt stress.^88^ Salt stress in tomato crop resulted in increased carotenoid‐related gene expression and thus carotenoid biosynthesis Similarly, the leaf lutein and *β*‐carotene contents of *Solanum villosum* and *Solanum nigrum* increased upon salt stress due to the up‐regulation of some biosynthesis genes.^89^, ^90^ This up‐regulation is probably related to the antioxidant effects of carotenoids which may be beneficial for defence mechanisms of the plants under stressful conditions. This is further reinforced by several literature data – for example, on sweet potato^91^, ^92^ – which indicate that plants with increased carotenoid contents (e.g., due to genetic engineering) have in general improved salt stress tolerance.

The somewhat contradictory data on photosynthetic pigments reflect differences in the applied salt stress treatments, in the specific salt tolerance of the tested plant species and cultivars, and in the species‐specific adaptive anatomical changes, such as leaves becoming thicker and narrower upon salt stress.^93^ This last alteration may indeed result in increased Chl and carotenoid content per leaf area and in a reduction on a per plant basis.

### Detection of the beneficial effects of halophytes by *in vivo* tests

Such a richness in substances with antioxidant, antiproliferative, anti‐inflammatory, and antibacterial activity underpins their possible therapeutic potential for various chronic conditions such as bacterial infections, cardiovascular diseases, neurodegenerative disorders, and cancer. In recent years, several studies have evaluated the beneficial effects of extracts produced from different halophytes through *in vivo* tests using human cell lines or animal models.

#### Anti‐inflammatory effects

Inflammation is a defensive response against various stimuli that involves the immune and vascular systems through the release of signalling molecules like cytokines, and changes in blood flow to affected areas. While acute inflammation is a beneficial reaction, chronic inflammation can be detrimental and can promote various health disorders, including autoimmune diseases, cardiovascular diseases, diabetes, bowel diseases, and cancer.

The anti‐inflammatory effect of bioactive compounds extracted from halophytes is often linked to the modulation of inflammatory pathways (mediated by cytokines such as interleukins (ILs), tumour necrosis factors (TNFs), and enzymes like cyclooxygenase 2 (COX‐2)), particularly in conditions related to oxidative stress and neuroinflammation. Alike, nitric oxide (NO) plays a dual role in inflammation, acting as both a pro‐inflammatory mediator and an anti‐inflammatory modulator. While NO is often associated with the promotion of inflammation through the activation of inflammatory pathways it can also contribute to the resolution of inflammation by promoting tissue repair and regulating immune cell activity, depending on the local environment and the stage of the inflammatory response.^94^ Halophytic plants have demonstrated the capacity to modulate NO production, making them promising candidates for therapeutic applications as anti‐inflammatory compounds. Table 1 reports examples on the anti‐inflammatory effects of several halophyte extracts.^95^, ^96^, ^97^, ^98^

**Table 1:** Anti‐inflammatory activity of halophytes' extracts

Plant species	Activity and results	Reference
*Salicornia europaea* ^a^	Anti‐neuroinflammatory and anti‐Parkinson's disease (PD)‐like effects on mice: extract was effective in attenuating the motor deficit in MPTP‐induced PD‐like mice; flavonoid irilin B effectively reduced the elevated expression of proinflammatory mRNA markers in lipopolysaccharide (LPS)‐stimulated cells.	Kim *et al*.^95^
*Salicornia europaea* ^a^	HT‐29 human cell line exposed for 24 h to the pro‐inflammatory cytochine TNF‐*α*: effective in decreasing the transcript levels of interleukin‐8 (IL‐8) in the tumour necrosis factor alpha (TNF‐*α*)‐treated cell also when applied at the lowest concentration, and in reducing cyclooxygenase 2 (COX‐2) expression even in the control cells, not treated with the pro‐inflammatory TNF‐*α* agent	Castagna *et al*.^96^
*Reaumuria vermiculata* ^b^	Anti‐inflammatory effects of dichloromethane extracts occurred in a dose‐dependent manner and achieved complete inhibition of nitric oxide (NO) release at the highest concentration tested (80 μg mL^−1^) in LPS‐stimulated RAW 264.7 murine macrophages. *Reaumuria vermiculata* also exhibited significant antioxidant activity in both *in vitro* and cell‐based assays, which further supports its therapeutic potential against oxidative stress‐related inflammation	Karker *et al*.^97^
*Polygonum maritimum* ^a^	Efficient suppression of NO production in LPS‐stimulated macrophages (RAW 264.7 murine line) by dichloromethane leaf extracts in a dose‐dependent manner when applied at concentrations ranging from 25 to 100 mg mL^−1^	Rodrigues *et al*.^98^

^a^ Validated as halophytes through eHALOPH database (https://ehaloph.uc.pt/);

^b^ Validated as halophyte through scientific literature, i.e., Karker *et al*.^97^

#### Anticancer properties

Cancer is a heterogeneous group of diseases characterized by the uncontrolled proliferation of malignant cells. According to the National Cancer Institute, global cancer incidence is projected to reach 29.9 million new cases per year by 2040, driven by population growth and ageing.^99^ Over the past two decades, significant advances have been made in cancer biology. However, despite these developments, chemotherapy continues to be one of the main therapeutic options. Unfortunately, its application is frequently associated with severe side effects which substantially limits treatment efficacy.

Given these challenges, there is a pressing need for novel anticancer therapies that are both effective and less toxic. In this context, natural compounds have gained increasing attention. As mentioned earlier, halophytes represent a promising reservoir of bioactive molecules. Several species from families such as Asteraceae, Amaranthaceae, Fabaceae, and Plumbaginaceae have demonstrated significant anticancer properties, exhibiting cytotoxic effects against aggressive cancer cell lines, including cervical cancer (HeLa), colorectal cancer (HCT‐116, DLD‐1, HT‐29), breast cancer (MCF‐7, MDA‐MB‐231), and liver cancer (HepG2). These halophyte‐derived bioactive compounds can modulate key oncogenic pathways, leading to apoptosis induction, cell cycle arrest, and inhibition of metastasis.^100^

According to the National Cancer Institute, crude extracts or fractions displaying half‐maximal inhibitory concentration (IC_50_) values below 30 μg mL^−1^ in preliminary assays are considered promising candidates for further research in anticancer drug development.^101^ Table 2 reports literature data on the anticancer effects of halophytes' extracts.^102^, ^103^, ^104^, ^105^, ^106^, ^107^, ^108^, ^109^, ^110^, ^111^

**Table 2:** Anticancer activity of halophyte extracts

Plant species	Activity and results	References
*Chenopodium ambrosioides* ^a^	Strong cytotoxic effects on MCF7 cells, with half‐maximal inhibitory concentration (IC_50_) values as low as 9.45 μg mL^−1^ after 24 h, inducing DNA fragmentation	Jia‐liang *et al*.^102^
*Suaeda fruticose* ^a^	Effective role against HCT116, DLD‐1, and HT‐29 colorectal cancer cells, with IC_50_ values of 17.2, 10, and 12 μg mL^−1^, respectively, causing cell‐cycle arrest in the G0–G1 phase and apoptosis induction	Saleh *et al*.^103^
*Achillea millefolium* ^a^	Significant activity of ethyl acetate extracts against HeLa and K562 cells (IC_50_ = 0.58 and 0.73 μg mL^−1^, respectively), with mechanisms involving apoptosis induction and cell‐cycle arrest in the G2/M phase	Abou Baker^104^
*Calystegia soldanella* ^a^	Cytotoxic activity of aqueous methanol extracts against HepG2 liver cancer cells, with an IC_50_ below 30 μg mL^−1^, inducing apoptosis and cell‐cycle arrest in the G0/G1 and S phases	Lee *et al*.^105^
*Cymodocea rotundata* ^a^	Promising activity against MG63 osteosarcoma cells, with an IC_50_ of 25.31 μg mL^−1^ for water leaf extracts	Abraham *et al*.^106^
*Melilotus indicus* ^a^	Cytotoxicity of methanol extracts against hepatocellular carcinoma (HepG2 and SNU‐182) cells, with IC_50_ values of 16.6 and 13.21 μg mL^−1^, respectively, via apoptosis induction and mitochondrial membrane potential loss	El‐Hafeez *et al*.^107^
*Eucalyptus camaldulensis* ^a^	Strong cytotoxicity of methanol, ethyl acetate, *n*‐butanol, and water extracts from leaves against MCF7 and MDA‐MB‐231, with IC_50_ values ranging between 4.9 and 26.7 μg mL^−1^	Hrubik *et al*.^108^
*Plantago major* ^a^	Inhibition of MCF7 cells by seed water extracts with an IC_50_ of 12 μg mL^−1^	Poor *et al*.^109^
*Echinochloa crus‐galli* ^a^	Inhibition of multiple cancer cell lines, including MCF7, HCT116, HeLa, and HepG2, by 70% ethanol extracts from grains, with IC_50_ values ranging from 4.2 to 18.9 μg mL^−1^	El Molla *et al*.^110^
*Lycium barbarum* ^a^	Cytotoxicity against glioblastoma stem‐like cells GSC‐3# and GSC‐12#, with IC_50_ values of 28 and 20 μg mL^−1^, respectively, by stem ethanol extracts	Zhu *et al*.^111^

^a^ Validated as halophytes through eHALOPH database (https://ehaloph.uc.pt/).

Further research is however essential to translate these findings into clinical applications. Additionally, *in vivo* studies and preclinical trials are required to assess their pharmacokinetics, bioavailability, and toxicity profiles. Moreover, an integrative approach combining computational modelling, high‐throughput screening, and metabolomic profiling may facilitate the identification of new lead compounds.

### Context‐dependent nutritional or anti‐nutritional metabolites

Despite the earlier‐described potential, halophytes also produce a variety of secondary metabolites that, while beneficial for plant survival, may pose nutritional challenges for human and animal consumption. Among these, the so‐called anti‐nutrients reduce the bioavailability of minerals, digestibility of proteins, and may also lead to toxicity. Anti‐nutritional factors such as phytates, tannins, alkaloids, saponins, oxalates, glucosinolates, lectins, cyanogenic glucosides, and protease inhibitors, can accumulate in the plant tissues at concentrations that deserve careful analysis. Their anti‐nutritional effect, though significant, is nevertheless context‐dependent, and such metabolites are also known for various health benefits in controlled dietary contexts.

#### Phytate

Phytate, or phytic acid, is widely recognized as a significant anti‐nutritional factor. In plants it serves as the primary storage form of P, particularly in seeds and grains^112^ and it is critical for plant development and stress resilience. Typically present in plant material in the form of a mixed salt known as phytin, it is commonly associated with Mg, K and Ca,^113^ depending on the species. Located in different parts of plant seeds such as the endosperm, germ or aleurone layer, it may be embedded with storage proteins or form electron dense, spherical structures, globoids, consisting mainly of phytate, protein and chelated ions (reviewed in Madsen and Brinch‐Pedersen^114^). Phytate is also present in leaves. There is considerable variation in the phytate content of leafy vegetables reported in the scientific literature, which is likely in part due to methodological issues, however it seems clear that phytate generally make up < 10% of total P in leaves.^115^

Phytates can bind to divalent cations like Zn, Fe, and Ca, forming soluble complexes in the acidic environment of the stomach. However, these complexes tend to precipitate at the physiological pH of the intestine and cannot be digested by human enzymes, thereby reducing the bioavailability of these minerals in the digestive tract.^116^ Mainly due to this chelating property, phytic acid is considered as one of the most effective anti‐nutrients in foods, and a cause of mineral deficiencies in animal and human nutrition.

Low‐income developing countries that primarily depend on grains and legumes as staple foods face a heightened risk of Zn deficiency or insufficiency.^117^ However, though phytates have been shown to reduce Zn bioavailability in adults, no significant effects have been observed in young children.^118^ Often, food rich in phytic acid also contains fermentable fibre that is able to acidify the caecum pH, thus favouring Fe absorption.^119^ Additionally, cell studies have shown that ascorbic acid can counteract the effect of phytic acid on Fe bioavailability, underlying the importance of the food matrix in influencing the impact of ingested phytates on mineral absorption.^120^ Given that the anti‐nutritional effect is context‐dependent, phytates have also been recognized for several positive roles in human nutrition,^116^, ^121^, ^122^, ^123^, ^124^, ^125^, ^126^, ^127^, ^128^, ^129^, ^130^, ^131^, ^132^, ^133^, ^134^ as reported in Table 3.

**Table 3:** Beneficial activities of phytates, tannins and oxalates

Compound	Effects observed	References
Phytates	Antioxidant properties, potential anticancer effects and various health benefits in controlled dietary contexts	López‐Moreno *et al*.^116^
	Reduced formation of reactive oxygen species (ROS) via Fenton reaction due to iron‐chelating ability	Silva and Bracarense^122^
	Decreased lipid peroxidation and scavenging of free radicals generated from the autooxidation of linoleic acid	Anekonda *et al*.^123^
	Mitigated formation of the deleterious advanced glycation end‐products (AGEs) in patients with type 2 diabetes mellitus	Sanchis *et al*.^124^
	Positive effects in various metabolic disorders, such as high triglyceride and cholesterol levels	Lee *et al*.^125^
	Positive effects in neurological diseases, including Alzheimer's disease	Anekonda *et al*.^123^
	Positive effects in neurological diseases, including Parkinson's disease	Chen *et al*.^126^
Tannins	Enhanced glucose absorption and inhibition of adipogenesis positioning them as potential therapeutic agents for the treatment of non‐insulin dependent diabetes mellitus	Muthusamy *et al*.^127^
	Cardioprotective effects of proanthocyanidins have been described and associated to antioxidative properties	Peluso and Serafini^128^
	Up‐regulation of a transcription factor involved in antioxidant defence systems	Shah *et al*.^130^
	Interference with the carcinogenesis process by lowering or inhibiting cytochrome P450 enzymes, and inducing glutathione‐s‐transferase, UDP and NAD(P)H‐quinone reductase activity, as well as due to their antioxidant, anti‐inflammatory and anti‐apoptosis activities	Kleszcz *et al*.^132^
	Improved cognitive functions in the elderly has been associated with intake of tannins‐rich foods	Mastroiacovo *et al*.^133^
Oxalates	Stimulation of chloride, water, and sodium absorption in the kidney, role in the production of hydrogen peroxide (H_2_O_2_) by oxalate oxidase, which can enhance the burst of phagocytes, and the formation of uracil acid, essential for RNA synthesis	Stepanova^134^

Phytates have been reported in various halophyte species,^135^ including glassworts (*Salicornia* sp.), seepweeds or seablite (*Suaeda* sp.), samphire (*Tecticornia* sp.), saltbush (*Atriplex* sp.), and quinoa (*Chenopodium quinoa*). The phytate content in halophytes varies significantly depending on species, growth conditions, and environmental stress factors. The phytate concentration of six *Tecticornia* species ranged between 6 and 46.2 mg g^−1^ dry weight (d.w.) and, except for one species, was lower than that of the common spinach (46.0 mg g^−1^ d.w.).^136^ Similarly, the phytate concentration of both seablite and saltbush was lower as compared to spinach, while seapurslane (*Sesuvium portulacastrum*) contained phytate concentration comparable to spinach (45.8 mg g^−1^ d.w.).^137^ Quinoa grains have been reported to contain moderately elevated phytate levels compared to commonly used grains. Polishing only removed a minor fraction of the phytate, suggesting a more even distribution of phytate in the seed compared to the cereals.^138^

#### Tannins

Tannins are a broad class of polyphenol compounds of high molecular weight (500–3000 Da) that play a crucial role in plant defence against herbivores and pathogens. These compounds are ubiquitously present in commonly consumed plant foods and are responsible for their astringent taste.^139^, ^140^ Tannins are classified into hydrolysable tannins (i.e., gallotannins and ellagitannins) and condensed tannins (flavanols, or proanthocyanidins, formed by polymers of catechin, epicatechin, gallocatechin, epigallocatechin, epicatechin‐3‐gallate and epigallocatechin‐3‐gallate).^141^ Their ability to form complexes with macromolecules like proteins and carbohydrates can hinder protein digestibility and nutrient absorption in animals and humans. Also, tannins negatively affect the activity of protein, lipid and carbohydrate digestive enzymes, such as tripsin,^142^ lipase,^143^ *α*‐amylase and glucoamylase.^144^ Moreover, tannins may chelate minerals such as Fe, Cu, and Zn, thus potentially reducing their absorption.^145^ Contradictory reports about a reduction in Fe bioavailability, serum Fe and haemoglobin in animals and humans are present in the literature (reviewed in Delimont *et al*.^146^). Usually, the concentrations of tannins typically consumed through a diverse diet are much lower than those used in supplementation. Moreover, the effects of tannins on Fe parameters have different causes (i.e., food matrix, presence of other bioactive compounds, timing of consumption, gender, dietary style) (reviewed in Petroski and Minich and Delimont *et al*.^121^, ^146^). This could explain why epidemiological studies show no correlation between tannin intake and Fe deficiency anaemia.

Despite the earlier described anti‐nutritional effects, tannins also exhibit several beneficial activities^147^ described in detail in Table 3.

Halophytes are known to contain tannins, which can vary in concentration depending on the species and environmental conditions. The concentration of hydrolysable tannins of six *Tecticornia* species collected in Western Australia ranged from 1.2 to 7.4 mg g^−1^ d.w., confirming the wide genotype‐dependent variation for these compounds.^136^ The same authors determined the concentration of hydrolysable tannins in the leaves of samphire and saltbush collected from three different sublocations, and found remarkable differences, mainly in samphire, where hydrolysable tannins ranged from 0.75 to 7.92 mg g^−1^ d.w., while the sublocation impacted less on tannin levels of saltbush (2.79–4.30 mg g^−1^ d.w.).^148^ The tannin concentration of the edible portion of *Salicornia europaea* cultivated under glasshouse conditions in Italy was about 0.3 mg g^−1^ of fresh weight (f.w.) and it was unaffected by the cultivation system, that is, monoculture *versus* consociation with tomato plants.^96^ A comprehensive analysis of bioactive metabolites present in 24 halophytes from coastal saline habitats of the Balkan Peninsula has been performed by Stanković *et al*.^149^ Significant variability in tannin concentrations has been observed across species, ranging from 0.05 mg mL^−1^ in *Halimione portulacoides* to 3.50 mg mL^−1^ in *Xanthium italicum*. However, the tannin levels in *Salsola kali*, the only species collected from three different countries, remained relatively consistent across all locations.

#### Oxalates

Oxalates are anti‐nutrient compounds derived from the binding of oxalic acid to minerals, forming salt crystals that can be water‐soluble (Na, K, and NH_4_^+^‐oxalates) or partially or completely water‐insoluble (Ca, Fe, Mg, and Zn‐oxalates).^150^ Notably, Ca oxalate crystals are common and appear to be formed in a regulated way with specialized purposes^151^ When ingested, unbound oxalates may chelate minerals and reduce their absorption and bioavailability, while, once absorbed, they can contribute to the formation of Ca oxalate kidney stones and potentially increase the risk of nephrotoxicity.^150^ Insoluble oxalates, being less bioavailable, pose less risks to human health and are generally excreted with the faeces. Indeed, when oxalate‐rich foods are consumed by persons with low‐Ca dietary uptake, higher risk of kidney stone formation was reported, probably due to higher absorption of unbound, soluble oxalate.^116^

Different from other anti‐nutrients, lower evidence of positive effects on human health have been reported for oxalates. Table 3 reports those effects.

Srivarathan *et al*.^137^ found that the oxalate content of sea purslane collected in Western Australia was almost as high as in spinach (38.5 and 40.4 mg g^−1^ d.w., respectively), that is considered an oxalate‐rich food. However, saltbush and seablite contained less oxalate on a d.w. basis, but their content was even higher than in spinach when reported on a f.w. basis. Differences in oxalate content ascribable to the growth location were observed in samphire (17.7–25.3 mg g^−1^ d.w.) and, even more, in saltbush (9.6–36.3 mg g^−1^ d.w.).^137^ Though no data on soil salinity and other soil attributes have been provided, this report presents interesting information about the interplay of climatic conditions, soil characteristics and ecotypes in determining the amount of oxalate in these species.

An interesting article by Barkla *et al*.^152^ draws attention to the issue of oxalate content in halophytes as a challenge to be addressed for the cultivation and commercial exploitation of these plants as food, as well as for the consumer's acceptance. Apart from technological processing like heating, fermenting, and blanching, that have the disadvantage of reducing the nutraceutical benefits provided by these plants, agronomic approaches seem to be very promising in achieving this goal. Oxalate content is indeed influenced by soil type, salinity of both soil and irrigation water, fertilization, and plant development stage.^153^ Among the agronomic practices, balancing N fertilization by increasing the NH_4_^+^/NO_3_^−^ ratio is a relatively easy method to lower oxalate concentration, also providing a reduction in shoot nitrate concentration, particularly under hydroponic/glasshouse production systems. The nitrifying activity of soil bacteria, that convert NH_4_^+^ to NO_3_^−^, makes it more difficult to use this approach in open field, unless N fertilizers containing nitrification inhibitors are used or halophytes are cultivated in highly acidic soils, that can naturally suppress nitrification.^154^, ^155^ However, evidence has been reported about a concomitant reduction in shoot biomass of ice plant (*Mesembryanthemum crystallinum*) and ruby saltbush (*Enchylaena tomentosa*)^62^ due to lowering the proportion of N provided as NO_3_^−^. Similarly, varying degrees of N‐dependent yield loss have been detected in different members of the *Portulaca oleracea* aggregate,^156^ emphasizing the importance of carefully selecting the optimal N source to maximize oxalate reduction while minimizing its impact on biomass production.

## PROTEINS

Protein quality refers to digestibility, amino acid profile, and bioavailability. Plant proteins supply all essential amino acids, are environmentally friendly, and are high‐quality sources for human consumption. Halophytes and glycophytes with enhanced salt tolerance activate a suite of proteins to maintain metabolic stability, regulate ion fluxes, synthesize compatible solutes, and scavenge ROS.^13^ These molecular adaptations enable them to survive, grow, and even thrive in environments where most plants would suffer significant damage.^157^ This section explores the molecular underpinnings of salt stress resistance in halophytes and salt‐tolerant glycophytes, shedding light on critical protein networks that contribute to plant resilience.

### Stress‐responsive proteins

Table 4 details the structural and functional characteristics of stress‐responsive proteins, together with gene expression and the main stress responsive pathways.

**Table 4:** Structural and functional characteristics, gene expression and stress responsive pathways of stress‐responsive proteins

1. Heat shock proteins (HSPs)	
Structural and functional characteristics	Gene expression and stress responsive pathways
HSPs are categorized based on their molecular weight, with each class performing specific functions related to protein stabilization and repair. Their major protective roles include: ‐ Protein homeostasis and refolding: HSP70 and HSP90 function as central regulators of the proteostasis network ‐ Protection of membranes and photosynthetic machinery ‐ Ion homeostasis and transport regulation ‐ Reactive oxygen species (ROS) detoxification and antioxidant defence ‐ Enhancement of metabolic and signalling pathways	HSP gene expression is tightly controlled by heat shock factors (HSFs), which recognize and bind to heat shock elements (HSEs) in the promoters of HSP genes. Under salt stress, protein misfolding and aggregation trigger the activation of HSFs, leading to a rapid up‐regulation of HSP expression. The activation of HSPs under salinity involves cross‐talk between multiple signalling pathways, including: Abscisic acid (ABA)‐dependent pathways – ABA accumulates under salt stress and promotes the expression of stress‐responsive HSPs; calcium signalling pathways – calcium ion (Ca^2+^) influxes induced by salt stress activate protein kinases that enhance HSP transcription; ROS signalling – elevated ROS levels act as secondary messengers in activating stress‐responsive genes, including HSPs.

2. Late embryogenesis abundant (LEA) proteins	
Structural and functional characteristics	Gene expression and stress responsive pathways
LEA proteins are typically highly hydrophilic and contain repetitive amino acid sequences that allow them to function as molecular shields, preventing the aggregation of other proteins under stress conditions. They can dynamically interact with other macromolecules, enhancing stress tolerance in plants. Their protective roles extend beyond simple osmoprotection and include: enhancing cellular homeostasis: by interacting with osmolytes such as proline and glycine betaine; reducing oxidative damage: some members of the LEA protein family have been shown to possess antioxidant properties, scavenging ROS generated under salt stress; assisting in metabolic adjustments: LEA proteins can modulate metabolic enzymes, allowing plants to redirect energy to pathways essential for stress survival.	The expression of LEA genes is regulated by multiple stress‐induced signalling pathways, including ABA‐dependent and ABA‐independent mechanisms. The accumulation of ABA under salt stress triggers transcription factors such as ABRE‐binding proteins (AREB/ABF), DREB (dehydration‐responsive element binding) proteins, and NAC transcription factors, which in turn activate LEA gene transcription.

#### Heat shock proteins (HSPs).

Heat shock proteins (HSPs) are a highly conserved group of molecular chaperones that play a critical role in plant stress tolerance, particularly under conditions of salinity, drought, and extreme temperatures. These proteins prevent protein misfolding, facilitating proper protein refolding, and promoting proteolysis of damaged proteins.^158^ Their essential role in stress adaptation makes them crucial for both halophytes and glycophytes with enhanced salt tolerance.

Halophytes exhibit a strong up‐regulation of HSP genes compared to glycophytes.^159^ However, some glycophytes, such as barley and tomato, have been found to also express high levels of HSPs under moderate salinity, suggesting that engineering HSP pathways in crops could enhance salt tolerance.^160^, ^161^ Given their crucial role in stress resilience, HSPs have been extensively studied for their potential applications in improving crop tolerance to salinity and other abiotic stresses. Strategies include: (i) genetic engineering^162^; (ii) breeding for stress‐tolerant crops using marker‐assisted selection targeting HSP‐related loci^163^; (iii) biostimulant development: application of HSP inducers (e.g., heat shock pretreatments) can prime plants against salt stress.^164^

#### Late Embryogenesis Abundant (LEA) Proteins.

Late embryogenesis abundant (LEA) proteins are a family of intrinsically disordered proteins that play a critical role in plant responses to abiotic stresses, particularly those associated with desiccation, osmotic imbalance, and high salinity.^165^, ^166^ Originally identified in the seeds of desiccation‐tolerant plants, LEA proteins have been found in various tissues, including leaves, roots, and stems of both halophytes and glycophytes with increased salt tolerance,^167^ and have gained interest in the development of genetically modified crops with enhanced salt and drought tolerance.^168^, ^169^, ^170^, ^171^ Overexpression of LEA genes in model and crop plants has demonstrated improvements in germination, biomass production, and overall stress resilience under high salinity conditions.

### Ion homeostasis proteins

The description of the structural and functional characteristics and of the gene expression and stress responsive pathways of ion homeostasis proteins and of osmoprotectant biosynthesis enzymes is reported in Table 5. Ion homeostasis is a critical factor for plant survival under salt stress, as excessive Na accumulation disrupts enzymatic processes, impairs osmotic balance, and leads to oxidative damage.^172^, ^173^ To counteract these effects, plants have developed specialized proteins that regulate Na sequestration, efflux, and K retention. These proteins include sodium/proton antiporters (NHX), salt overly sensitive (SOS) pathway components, and K transporters (HKT and AKT), all of which contribute to maintaining ionic balance and ensuring cellular function under saline conditions.^174^

**Table 5:** Structural and functional characteristics, gene expression and stress responsive pathways of ion homeostasis proteins and osmoprotectant biosynthesis enzymes

Ion homeostasis proteins	
1. Proteins sodium/Proton antiporters (NHX)	
Structural and functional characteristics	Gene expression and stress responsive pathways
Sodium ion (Na^+^) sequestration and detoxification: NHX transporters prevent excessive cytoplasmic Na^+^ accumulation by compartmentalizing it in vacuoles. Osmotic balance regulation: accumulated Na^+^ in vacuoles helps maintain osmotic pressure, facilitating water uptake and retention. pH homeostasis and enzymatic stability: NHX‐mediated ion exchange contributes to cytoplasmic pH regulation, ensuring optimal enzymatic activity.	NHX expression and activity are tightly controlled by various stress‐responsive pathways, including: calcium signalling: increased calcium ion (Ca^2+^) levels under salt stress activate protein kinases that modulate NHX activity; abscisic acid (ABA) signalling: ABA accumulation enhances NHX gene transcription, linking ion homeostasis with broader stress responses. Post‐translational modifications (PTMs): phosphorylation and ubiquitination fine‐tune NHX activity, optimizing its function under variable salinity conditions.

2. Salt overly sensitive (SOS) pathway proteins	
Structural and functional characteristics	Gene expression and stress responsive pathways
Salt stress triggers a Ca^2+^ influx, which is detected by SOS3. SOS3 activates SOS2, forming a kinase complex that phosphorylates target proteins. SOS2 phosphorylates and activates SOS1, a plasma membrane Na^+^/H^+^ antiporter, which expels excess Na^+^ from cells.	Transcriptional regulation: salt‐responsive transcription factors, including DREB, MYB, and NAC, enhance SOS gene expression; hormonal interactions: ABA signalling modulates SOS pathway activity, integrating salt tolerance with drought responses. Active Na^+^ exclusion: prevents toxic Na^+^ buildup in the cytoplasm; ion transport coordination: interacts with NHX antiporters and potassium ion (K^+^) transporters to maintain ionic balance; enhanced stress perception: links ion homeostasis with broader stress signalling networks.

3. Potassium transporters (HKT and AKT)	
Structural and functional characteristics	Gene expression and stress responsive pathways
HKT transporters (high‐affinity K^+^ transporters): HKT1‐type transporters selectively retrieve Na^+^ from the xylem, reducing sodium transport to leaves; HKT proteins regulate K^+^/Na^+^ selectivity, preventing ionic toxicity in root and shoot tissues; AKT transporters (arabidopsis K^+^ transporters): AKT1 facilitates K^+^ uptake, maintaining intracellular potassium levels; regulated by CBL‐CIPK (Calcineurin B‐Like/CBL‐Interacting Protein Kinases) networks, ensuring adaptive ion transport; role of potassium transporters in salt tolerance; prevent excessive K^+^ loss: stabilizes cellular osmotic conditions; optimize metabolic activity: supports photosynthesis and respiration; enhance adaptive stress signalling: modulates hormonal responses to salinity.	Together, NHX antiporters, the SOS pathway, and K^+^ transporters form an interconnected network that regulates ion fluxes, ensuring plant survival under high salinity. This integrated system enables: efficient Na^+^ exclusion (SOS1) and compartmentalization (NHX). Sustained K^+^ uptake and retention (HKT, AKT). Cross‐talk between stress signalling pathways and metabolic regulation. Understanding and enhancing these ion homeostasis mechanisms through genetic engineering and breeding strategies offers promising approaches to developing salt‐tolerant crops capable of thriving in saline environments.

Osmoprotectant biosynthesis enzymes	
1. Proline	
Structural and functional characteristics	Gene expression and stress responsive pathways
Osmotic regulation: accumulation of proline reduces water potential, allowing cells to retain water and sustain turgor pressure. Protein and membrane stabilization: protects enzymes and lipid bilayers from salt‐induced damage. Reactive oxygen species (ROS) detoxification: acts as an antioxidant, neutralizing free radicals generated by oxidative stress. Energy source during recovery: degradation of stored proline provides energy when stress conditions subside.	The biosynthesis of proline occurs via the glutamate and ornithine pathways, with the glutamate pathway being the predominant route under salt stress: *Δ* ^1^‐pyrroline‐5‐carboxylate synthetase (P5CS): catalyses the conversion of glutamate to pyrroline‐5‐carboxylate (P5C), a key intermediate in proline biosynthesis. Pyrroline‐5‐carboxylate reductase (P5CR): converts P5C into proline, finalizing the biosynthetic process. Regulation of proline biosynthesis under salt stress: abscisic acid (ABA)‐dependent pathways enhance the transcription of P5CS and P5CR, leading to increased proline accumulation; DREB and NAC transcription factors regulate proline biosynthetic genes in response to salt stress; feedback inhibition mechanisms prevent excessive proline accumulation, maintaining metabolic balance.

2. Glycine betaine (GB)	
Structural and functional characteristics	Gene expression and stress responsive pathways
Maintains membrane integrity: protects phospholipid bilayers from ion‐induced damage. Enhances photosynthesis: prevents salt‐induced degradation of photosystem II (PSII) and Rubisco enzymes. Improves enzyme stability: maintains the function of macromolecules under osmotic stress. Supports ion homeostasis: helps balance Na^+^ and K^+^ levels in plant cells.	GB is synthesized from choline via a two‐step oxidation process: Choline monooxygenase (CMO): converts choline into betaine aldehyde. Betaine aldehyde dehydrogenase (BADH): converts betaine aldehyde into GB. Transcriptional activation of CMO and BADH genes occurs under saline conditions. ABA and jasmonic acid (JA) signalling pathways contribute to GB accumulation. Epigenetic modifications (e.g., DNA methylation and histone acetylation) influence GB biosynthetic gene expression under prolonged salt stress.

3. Trehalose	
Structural and functional characteristics	Gene expression and stress responsive pathways
Protects proteins and membranes: stabilizes macromolecules against salt‐induced denaturation. Regulates carbohydrate metabolism: maintains cellular energy homeostasis under stress conditions. Acts as a signalling molecule: modulates stress‐responsive pathways, including ABA and sugar metabolism pathways. Reduces oxidative stress: enhances ROS scavenging by interacting with antioxidant defence proteins.	Trehalose biosynthesis occurs through a two‐step enzymatic process: trehalose‐6‐phosphate synthase (TPS): catalyses the formation of trehalose‐6‐phosphate (T6P) from glucose‐6‐phosphate and UDP‐glucose; trehalose‐6‐phosphate phosphatase (TPP): converts T6P into trehalose, which can be stored or used for osmoprotection; ABA signalling enhances TPS expression, linking trehalose accumulation to drought and salt stress responses; SnRK1 (Sucrose Non‐Fermenting 1‐Related Protein Kinase 1) signalling pathway modulates trehalose metabolism to balance energy production and stress responses; trehalose acts as a feedback regulator of carbohydrate metabolism.

#### Proteins sodium/proton antiporters (NHX)

NHX transporters are membrane‐bound proteins that facilitate the sequestration of excess Na into vacuoles, preventing cytoplasmic toxicity. These proteins function by exchanging Na for protons (H^+^), utilizing the electrochemical gradient established by H^+^‐ATPases and H^+^‐pyrophosphatases (H^+^‐PPases).^175^, ^176^, ^177^

#### Salt overly sensitive (SOS) pathway proteins

The SOS pathway is a key regulatory system that ensures Na efflux and prevents its toxic accumulation in the cytoplasm. This pathway consists of three core proteins: SOS1 (Na^+^/H^+^ antiporter), SOS2 (protein kinase), and SOS3 (calcium‐binding sensor protein).^178^, ^179^, ^180^, ^181^

#### Potassium transporters (HKT and AKT)

Maintaining a high K/Na ratio is essential for plant metabolism, as K plays a key role in enzymatic activity, turgor maintenance, and electrical signalling. Under salt stress, K transporters prevent K leakage and ensure adequate K uptake.^182^, ^183^, ^184^, ^185^

### Osmoprotectant biosynthesis enzymes

Osmoprotectants are low‐molecular‐weight organic compounds that play a critical role in protecting plants against salt stress. These molecules help maintain cellular osmotic balance, stabilize macromolecules, and mitigate oxidative damage. Among the most important osmoprotectants involved in plant salt tolerance are proline, glycine betaine, and trehalose, which act as osmolytes, antioxidants, and metabolic regulators.^186^, ^187^, ^188^, ^189^ Plants rely on specific biosynthetic enzymes to synthesize these compounds under stress conditions. The up‐regulation of proline biosynthesis enzymes, glycine betaine synthesis pathways, and trehalose metabolism enzymes contributes to osmotic adjustment and enhances plant resilience to salinity.

#### Proline.

Proline is one of the most well‐characterized osmoprotectants in salt‐tolerant plants: it functions as an osmolyte, molecular chaperone, and ROS scavenger.^190^, ^191^, ^192^, ^193^

#### Glycine betaine (GB).

Glycine betaine (GB) is a quaternary ammonium compound that serves as an osmoprotectant and enzyme stabilizer in plants exposed to high salinity. Unlike proline, which functions primarily as an osmolyte, GB plays a more structural role by stabilizing proteins, membranes, and photosynthetic complexes under salt stress.^194^, ^195^, ^196^, ^197^, ^198^

#### Trehalose metabolism.

Trehalose is a non‐reducing disaccharide that serves as an osmoprotectant and metabolic regulator in salt‐stressed plants. Although traditionally associated with desiccation tolerance, trehalose has gained attention for its role in enhancing salt stress tolerance in both halophytes and glycophytes.^199^, ^200^, ^201^, ^202^, ^203^, ^204^

### Antioxidant defence proteins

Table 6 reports the structural and functional characteristics, gene expression and stress responsive pathways of antioxidant defence proteins and signal transduction proteins. Salt stress leads to excessive production of ROS, such as superoxide radicals (O_2_^−**·**^), hydrogen peroxide (H_2_O_2_), and hydroxyl radicals (OH^**·**^). While ROS play a role in stress signalling at low concentrations, their excessive accumulation causes oxidative damage to lipids, proteins, and nucleic acids.^205^, ^206^, ^207^, ^208^, ^209^ To counteract this, salt‐tolerant plants activate a robust antioxidant defence system, composed of enzymatic antioxidants, non‐enzymatic antioxidants, and redox regulatory proteins.

**Table 6:** Structural and functional characteristics, gene expression and stress responsive pathways of antioxidant defence proteins

Antioxidant defense proteins	
1. Superoxide dismutase (SOD)	
Structural and functional characteristics	Gene expression and stress responsive pathways
Prevents oxidative damage: converts the highly reactive superoxide radical into a less toxic form (hydrogen peroxide (H_2_O_2_)); regulates cellular redox state: helps maintain oxidative balance within chloroplasts, mitochondria, and cytoplasm; protects metabolic processes: reduces reactive oxygen species (ROS)‐induced damage to photosynthetic and respiratory enzymes. Types of SOD in plants: Cu/Zn‐SOD: found in the cytosol, chloroplasts, and peroxisomes, playing a central role in stress responses; Mn‐SOD: localized in mitochondria, protecting cellular respiration from oxidative stress; Fe‐SOD: present in chloroplasts, where it prevents ROS accumulation during photosynthesis.	Abscisic acid (ABA) and ethylene signalling pathways induce SOD gene expression in response to salinity. MYB and WRKY transcription factors enhance the transcription of SOD genes under oxidative stress conditions. Epigenetic modifications, such as DNA methylation and histone acetylation, influence SOD activity in long‐term stress adaptation

2. Catalase (CAT)	
Structural and functional characteristics	Gene expression and stress responsive pathways
Prevents oxidative stress by breaking down excess H_2_O_2_; maintains cellular homeostasis: ensures redox balance in peroxisomes, where H_2_O_2_ is generated; protects photosynthetic machinery: reduces oxidative damage to chloroplast components, preserving photosynthetic efficiency.	Increased expression of CAT genes (CAT1, CAT2, CAT3) in response to salt stress. Interaction with ROS signalling pathways, modulating redox‐sensitive transcription factors. Phosphorylation events regulate CAT activity, adjusting its function based on ROS levels.

3. Ascorbate–glutathione (AsA–GSH) cycle enzymes	
Structural and functional characteristics	Gene expression and stress responsive pathways
Reduces oxidative stress: neutralizes H_2_O_2_ and prevents ROS‐induced damage. Regulates stress signalling: modulates ABA, ethylene, and salicylic acid pathways involved in salt stress responses. Supports chloroplast function: maintains photosynthetic efficiency under high salinity.	Up‐regulation of APX, GR, and DHAR genes in salt‐tolerant plants. Interplay with hormonal signalling (ABA, auxins, salicylic acid), fine‐tuning antioxidant responses. Post‐translational modifications (e.g., phosphorylation, S‐nitrosylation) regulate enzyme activity and response dynamics.

The superoxide dismutase (SOD), catalase (CAT), and ascorbate–glutathione (AsA–GSH) cycle enzymes work in coordination to scavenge ROS, maintaining redox homeostasis and ensuring plant survival under saline conditions.^210^, ^211^ SOD is the first line of defence against oxidative stress, catalysing the dismutation of O_2_^−**·**^ into H_2_O_2_ and molecular oxygen (O_2_). This step is crucial for controlling ROS levels before they cause cellular damage.^212^, ^213^, ^214^ CAT is a crucial enzyme responsible for the detoxification of H_2_O_2_ by converting it into water and oxygen. This prevents the accumulation of toxic levels of H_2_O_2_, which can trigger lipid peroxidation and cell death.^215^, ^216^ The AsA–GSH cycle is one of the most important redox buffering systems in plants. It involves a series of enzymatic reactions that detoxify H_2_O_2_ while regenerating key antioxidants, such as ascorbate (vitamin C) and glutathione (GSH).^217^, ^218^, ^219^ The coordination between SOD, CAT, and the AsA–GSH cycle ensures an effective antioxidant defence system in plants exposed to high salinity.^210^, ^220^, ^221^ Together, these proteins: (i) prevent oxidative damage to proteins, lipids, and DNA; (ii) modulate ROS signalling for stress adaptation and response coordination; (iii) maintain metabolic stability, ensuring proper cellular function under prolonged stress. Salt‐tolerant plants often exhibit higher basal levels of antioxidant enzyme activity compared to salt‐sensitive glycophytes.^222^ Not surprisingly, salt stress influences the ascorbic acid contents of crops.^223^

## AROMA AND VOCs

Several plants used by the food, beverage, pharmaceutical or perfume industry produce high added value aroma and volatile organic compounds (VOCs).^224^ VOCs are small hydrophobic molecules with low molecular mass, high vapour pressure, low boiling point that can readily evaporate and diffuse through heterogeneous mixtures of solids, liquids and gasses, providing typical smell or fragrance.^225^ Plant VOCs belong to a broad range of chemical classes such as isoprenoids, phenylpropanoids, benzenoids, fatty acid derivatives, and amino acid derivatives. Isoprenoids mainly include terpenes (e.g., hemiterpenes, homoterpenes, monoterpenes, and sesquiterpenes) and volatile carotenoid derivatives (e.g., C13‐norisoprenoids^226^). VOCs play a key role in plant protection and plant–environment interactions and can have hormone or hormone‐like function in regulating certain aspects of plant development and signals (e.g., ethylene, methyl jasmonate, and methyl salicylate). Essential oils are mixtures of VOCs traditionally obtained by steam distillation of fruits, flowers, leaves, stems, seeds, aromatic woods, and roots. The main components of essential oils are hydrocarbons, including terpenes and their oxygenated derivatives, alcohols, aldehydes, ketones, and phenylpropanoids.^227^ These molecules provide health‐promoting biological activities that go beyond their role as aromas and natural preservatives and can be exploited to develop functional foods and diet supplements.^227^ Essential oils may also have antibacterial, antiviral, antifungal, antioxidant and anti‐inflammatory effects and are thus mostly used for respiratory tract infections. They may also affect brain function by decreasing mental fatigue, inducing relaxation, this way positively affecting cognitive performance and mood.^228^ The antibacterial property of some plant VOCs may be used by the food industry for preservation purposes. In addition, those plants characteristic aroma and fragrance might make them ideal spices, herbs or food additives.

Halophytes produce essential oils that contribute not only to their aroma and flavour but exhibit antibacterial, antifungal, antitumour, antioxidant, and spasmolytic activities.^229^, ^230^ Although their flavouring and health promoting effects, as well as their role in plant defence mechanisms against biotic stresses are well known, less information is available on the effect of abiotic stressors on their synthesis.^80^ However, scientific literature clearly indicates that the increased release of VOCs upon salt stress may play a role in signalling neighbouring plants, thereby priming them for stress tolerance.^80^, ^231^ Deriving general conclusions on how salinity influences the quantity and quality of VOCs remains challenging due to several factors, including the high chemical diversity of these compounds, their biological roles, the complexity of their biosynthetic pathways, and the fact that their production can be either constitutive or stress‐induced, leading to contradictory literature data.^232^

Salt stress can lead to changes in both the quantity and composition of VOCs. The influence of salt stress on the VOCs synthesis of plants is probably influenced by the tolerance of the given species or cultivar, the type, length and concentration of the treatment, various other external environmental conditions as well as the developmental stage of the plants. *Crithmum maritimum* L., an aromatic and edible halophyte from the Apiaceae family with economic and medicinal potential due to its edible properties and strong scent,^233^ showed as major classes of compounds monoterpene hydrocarbons, oxygenated monoterpenes, and phenylpropanoids.^234^ The ability of *Crithmum maritimum* to tolerate nutrient deprivation in combination with varying saline levels (ranging from 0.5 to 500 mmol L^−1^ NaCl, prepared using 5% and 20% Hoagland's solution) has been investigated by Castillo *et al*.^235^: under these conditions, the leaf concentrations of five terpenes (*α*‐terpinene, *p*‐cymene, limonene, *β*‐phellenadrene and *α*‐pinene) were found to be 50% and 76% lower at 500 mmol L^−1^ NaCl than under freshwater conditions. Additionally, the concentrations of these five terpenes, along with *β*‐myrcene and thymol methyl ether, were 30% and 50% lower in plants treated with low nutrient concentration compared to the controls. This opposite response to nutrient availability at higher salinities may be explained by the down‐regulation of the antioxidant defence systems, including terpenes, under nutrient deficiency and freshwater conditions, as well as up‐regulation due to salinity stress, particularly in nutrient‐limited plants.^236^ The edible halophyte *Eryngium maritimum* L. (sea holly, Apiaceae family) is known for its significant content of phenolic acids, flavonoids^237^ and essential oils extracted from its aerial parts and roots.^238^ Particularly, essential oils from aerial parts of Corsican and Sardinian populations primarily contain germacrene D (33.9%), 4*βH*‐cadin‐9‐en‐15‐al (26.1%), 4*βH*‐cadin‐9‐en‐15‐ol (9.5%) and 4*βH*‐muurol‐9‐en‐15‐al (5.2%), while those from the roots were mainly consisting of 2,4,5‐trimethylbenzaldehyde (39.8%), 2,3,6‐trimethylbenzaldehyde (29.0%), *α*‐muurolene (23.5%), trimethylbenzaldehyde, *γ*‐guaiene (40.2%), and germacrene D (10.6%).^239^ The ecotype significantly influences the chemical composition of essential oils extracted from air‐dried fruits of five Tunisian *Eryngium maritimum* populations with monoterpene hydrocarbons (germacrene B and D, ranging from 39.67% to 54.25%) and oxygenated sesquiterpenes (15‐hydroxy‐*α*‐muurolene, ranging from 28.27% to 32.35%) as the dominant compounds.^238^ The succulent halophyte golden samphire *Inula crithmoides* L. (syn. *Limbarda crithmoides* (L.) Dumort.) has edible young leaves characterized by high essential oil contents. These predominantly contain monoterpene hydrocarbons (with *α*‐pinene as the most abundant component, followed by *α*‐ and *β*‐phellandrene), sesquiterpene hydrocarbons (e.g., germacrene D), and oxygenated sesquiterpenes (e.g., *t*‐cadinol).^240^ More recent studies on an accession from the Aeolian Islands (Sicily, Italy) revealed that monoterpene hydrocarbons are the main volatile class (45.2% of the essential oil), with *β*‐myrcene as the most abundant component, followed by *p*‐cymene, *β*‐phellandrene, *α*‐pinene and camphene.^241^ Oxygenated monoterpenes, with thymol acetate as the main component, were the second most abundant class, while sesquiterpene hydrocarbons and oxygenated sesquiterpenes were present only in trace amounts.^241^ A recent study demonstrated that salinity affects the sensory quality traits of date palm (*Phoenix dactylifera* L.) fruits, including texture, flavour, aroma, taste, colour, and appearance in five different varieties^242^ Specifically, the Ajwa‐tul‐Madinah, Fard and Khalas varieties received lower appreciation in panel tests at electrical conductivity (EC) of 15 dS m^−1^ compared to the same varieties available on the market, in terms of flavour and aroma. In contrast, the Lulu and Sukkari varieties maintained similar appreciation levels in panel tests at both EC 15 dSm^−1^ and in market‐available samples. This suggests that each variety responds differently to salinity, highlighting the potential for targeted genotype selection.^242^

## PROSPECTS AND KNOWLEDGE GAPS

Recent advancements in plant biotechnology provide a highly controlled platform for studying and manipulating plant responses to salinity in a consistent and reproducible manner. *In vitro* methodologies facilitate the rapid screening of large plant populations, and are well‐suited for genetic transformation and metabolic engineering, supporting the amplification of naturally occurring beneficial compounds and offering opportunities to introduce novel traits through genetic and metabolic engineering.^243^ Traditional plant breeding approaches incorporate techniques like protoplast fusion and somaclonal variation to utilize existing genetic diversity or create new genetic variability. When combined with elicitation studies, plant tissue culture serves as a valuable tool for investigating the impact of salinity on plant nutritional quality, as controlled exposure to NaCl enables researchers to analyse its effects on plant metabolic pathways.^244^, ^245^ New genomic techniques (NGTs) like CRISPR/Cas9 have opened new avenues for enhancing both primary nutrients and secondary metabolites in crops through the precise target of genes associated with nutrient biosynthesis, creating biofortified crops to address global nutrient deficiencies. These advancements highlight the transformative potential of genetic engineering to create nutrient‐rich, health‐promoting, and sustainable crops.^246^ In addition, nanotechnology provides a sustainable and advanced approach to enhancing plant growth and metabolite production. Nanoparticles can activate plant defence pathways as elicitors or be engineered to deliver specific molecules or genes directly into plant cells, inducing target compound synthesis and enabling precise genetic modifications. Integrating nanoparticle technology with elicitation strategies and metabolic engineering holds great promise for enhancing the nutritional quality of plants.^247^ Moreover, plant microbiome engineering (PME) offers a promising avenue for enhancing the nutritional quality of crops by leveraging the interactions between plants and their associated microbial communities. By tailoring microbial consortia to specific crops and environmental conditions, PME holds the potential to sustainably improve both the nutritional content and overall productivity of plants.^248^

As soil salinity continues to threaten global agricultural productivity, understanding the molecular mechanisms underlying salt tolerance is essential for developing resilient crops. The integration of *in vitro* manipulation techniques presents a sustainable approach to enhancing the nutritional value of salt‐tolerant plants, unlocking their potential as a rich source of essential nutrients. Despite their significance, studies exploring the application of such techniques specifically in salt‐tolerant species remain limited, leaving a substantial knowledge gap in optimizing their nutritional profiles. Advancing this area of research could address critical food security challenges while enabling the productive use of saline and marginal landscapes for agriculture, as summarized in Fig. 2.

**Figure 2:**
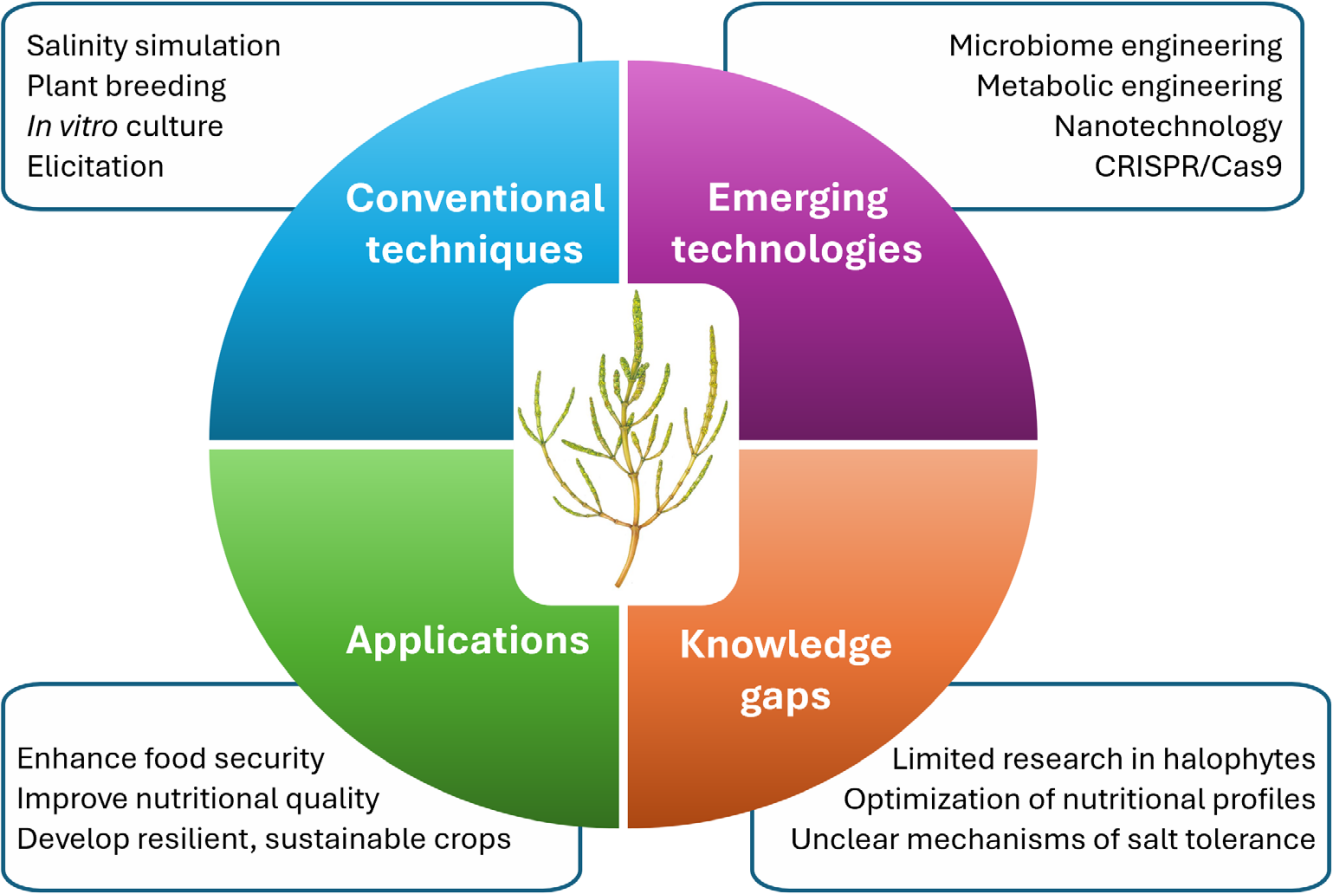
Prospects and knowledge gaps in the agricultural use of halophytes.

We believe that a multidisciplinary approach involving molecular biology, systems biology, plant breeding, agronomy, and digital agriculture will be crucial in ensuring sustainable food production in saline‐prone regions. By addressing these research gaps and leveraging cutting‐edge technologies, the scientific community can unlock the full potential of salt‐tolerant plants, ensuring agricultural sustainability and global food security in the face of increasing soil salinization.

## Data Availability

Data sharing not applicable to this article as no datasets were generated or analysed during the current study.
